# Increasing temperatures reduce invertebrate abundance and slow decomposition

**DOI:** 10.1371/journal.pone.0259045

**Published:** 2021-11-10

**Authors:** Laura L. Figueroa, Audrey Maran, Shannon L. Pelini

**Affiliations:** 1 Harvard Forest, Harvard University, Petersham, Massachusetts, United States of America; 2 Department of Biological Sciences, Bowling Green State University, Bowling Green, Ohio, United States of America; Helmholtz Zentrum Munchen Deutsches Forschungszentrum fur Umwelt und Gesundheit, GERMANY

## Abstract

Decomposition is an essential ecosystem service driven by interacting biotic and abiotic factors. Increasing temperatures due to climate change can affect soil moisture, soil fauna, and subsequently, decomposition. Understanding how projected climate change scenarios will affect decomposition is of vital importance for predicting nutrient cycling and ecosystem health. In this study, we experimentally addressed the question of how the early stages of decomposition would vary along a gradient of projected climate change scenarios. Given the importance of biodiversity for ecosystem service provisioning, we measured the effect of invertebrate exclusion on red maple (*Acer rubrum*) leaf litter breakdown along a temperature gradient using litterbags in warming chambers over a period of five weeks. Leaf litter decomposed more slowly in the warmer chambers and in the litterbag treatment that minimized invertebrate access. Moreover, increasing air temperature reduced invertebrate abundance and richness, and altered the community composition, independent of exclusion treatment. Using structural equation models, we were able to disentangle the effects of average air temperature on leaf litter loss, finding a direct negative effect of warming on the early stages of decomposition, independent of invertebrate abundance. This result indicates that not only can climate change affect the invertebrate community, but may also directly influence how the remaining organisms interact with their environment and their effectiveness at provisioning ecosystem services. Overall, our study highlights the role of biodiversity in maintaining ecosystem services and contributes to our understanding of how climate change could disrupt nutrient cycling.

## Introduction

In light of current and projected increases in global average temperature, understanding the relative contribution of various biotic and abiotic factors on ecosystem service provisioning under various climate change scenarios becomes of utmost importance [1, 2]. The relationship between decomposition and climate is interdependent, with climate being a primary predictor of decomposition in many ecosystems [3–5]. However, recent research illustrates that the predictive power of climate on decomposition may be overestimated. For example, Bradford et al. propose that other variables, including top-down predator control of the invertebrate community and nitrogen availability, could account for more variation in decomposition rates than climate [6]. In addition, studies of decomposition and climate change are often confounded, largely due to differences in experimental approaches [7].

Environmental conditions and biotic communities shape complex ecological processes such as decomposition. Alongside climate, soil invertebrates are key drivers of decomposition rates across ecosystems [8, 9], as are the interactions between temperature, moisture, and soil fauna [10–12]. How these interactions between biotic and abiotic variables will be affected by warming is not entirely known as different leaf decomposition rates have been reported in response to warming. While many studies find increased decomposition as a direct response to warming [13–16], others have found no net change when warming impairs detritivore-induced decomposition [17], or even found markedly reduced decomposition as a product of experimental warming [18]. Particularly when warming results in moisture-limiting conditions, decomposition can be reduced substantially [19]. It is necessary to understand how biotic variables can mediate the effects of global change [20], and test under what conditions the prediction that decomposition rates increase with global climate change is valid [8].

Climate change can result in a substantial loss of biodiversity [21], including in soil ecosystems. The functional diversity of the decomposer community plays an important role in carbon and nitrogen cycling across ecosystems [22], as reduced soil biodiversity effectively limits decomposition and nutrient cycling [23]. Many macroinvertebrate taxa are known to increase decomposition rates; examples include earthworms [24], springtails [25], ants [18], and even spiders [26, 27]. Studies have tested the effect of temperature along a geographic temperature gradient [19, 28, 29], resulting in replicates that are subject to differences in precipitation, sunlight hours, invertebrate colonization, among other variables. There is a clear need for studies that minimize confounding factors that could affect litter decomposition in climate change studies [7] and explicitly measure soil communities, temperature, and moisture [30].

Given the magnitude of current and predicted climate change impacts on ecosystem functioning, understanding how fundamental ecosystem services such as decomposition will respond to warming is of great importance. Exploring ecological dynamics in their complexity by including biotic and abiotic components increases our ability to predict future changes. We deployed litterbags in experimental warming chambers that increased air temperature encompassing the range of projected climate change warming scenarios [2]. We evaluated 1) the interactive effect of invertebrate exclusion, average air temperature, and soil moisture on invertebrate abundance, richness, and community composition, and 2) whether these variables directly or indirectly drove leaf litter loss.

## Materials and methods

### Study site

**Fig 1:**
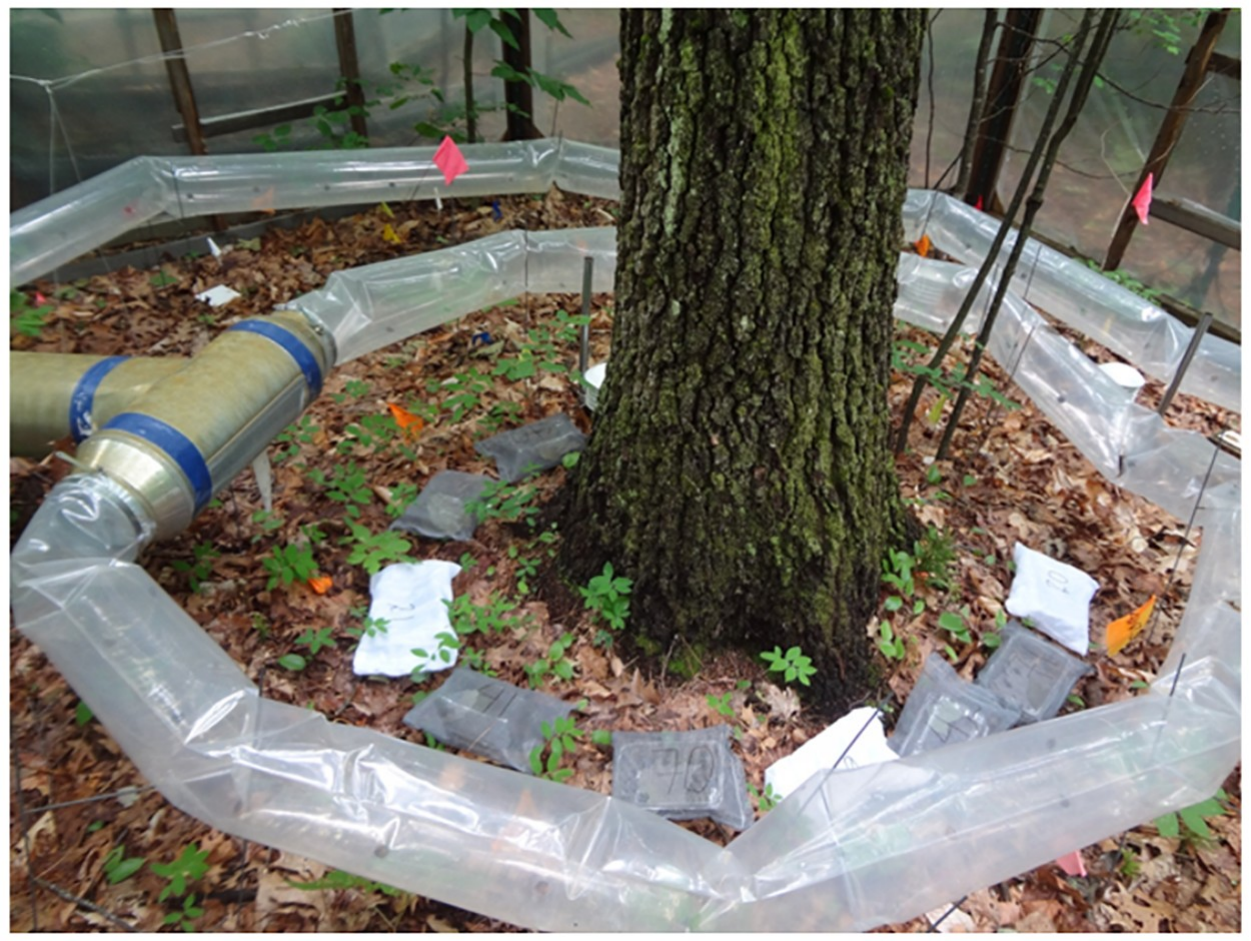
Litterbags placed in the Harvard Forest warming chambers. Different mesh sizes were used to control the size of the invertebrate community accessing the bags (‘invertebrate exclusion treatments’).

We conducted the experiment at Harvard Forest in Petersham, Massachusetts during the summer of 2014. Open-top warming chambers (5 m diameter) were used to regulate warming through forced air movement ([31] Fig 1). Ten warming chambers, each with a specific warming scenario were used: 0°C, 1.5°C, 2°C, 2.5°C, 3°C, 3.5°C, 4°C, 4.5°C, 5°C, and 5.5°C above ambient temperature [2]. The warming chambers had been operating for five years when we conducted this experiment. We measured hourly air temperature, organic soil temperature, and inorganic soil temperature each with two sensors, and soil moisture with one sensor for the duration of the experiment. The soil moisture sensors were at a 2 cm depth, the organic soil temperature sensors were at a 2 cm depth, the inorganic soil temperature sensors were at a 6 cm depth, and the air temperature sensors were approximately 50 cm from the ground. We used the mean value from the sensor readings from the day of deployment until the day of collection for each variable in subsequent statistical analyses. As Harvard Forest Investigators, we were authorized to use and collect on their property. No permits were required.

### Decomposition and invertebrate exclusion experiment

We measured decomposition in litterbags designed to exclude litter invertebrate fauna based on size. Three types of litterbags (‘invertebrate exclusion treatment’) were constructed: nylon micro-mesh of ~10x10 μm^2^ holes, fiberglass meso-mesh of 2.5x2.5 mm^2^ holes, and fiberglass macro-mesh of 1x1 cm^2^ holes which encompass the size of all major litter invertebrates known from this system (Pelini, personal observation). The bags were sewn from 47x28.5 cm pieces of material. We placed modified plastic strawberry containers in the bags to standardize the structure without reducing access to the litter (Fig 1). We modified the plastic containers to improve permeability by removing the top and cutting holes on the bottom of each container (S1 Fig). We used red maple leaves (*Acer rubrum*) as the decomposition substrate because it dominates the forests where the experiment was conducted, and is very common throughout eastern North American forests [32]. Additionally, red maple can decompose quickly [33, 34], thus enabling the assessment of early decay dynamics. We collected healthy fresh rather than senesced leaves directly from living trees to standardize the initial conditions of the substrate, an experimental approach that can be used to compare among treatments [35]. The same day of collection, the leaves were dried in an industrial oven at 70º C for 24h to measure the initial dry-weight (*M*_*0*_). Within each of the ten warming chambers, three replicates of each litterbag size were placed on the forest floor on top of existing maple litter for a total of nine bags per chamber and thus 90 litterbags total. We deployed the bags on June 27, 2014, with an initial weight of 0.9 ± 0.05 g of litter. We collected the litterbags over three collection dates, taking one litterbag replicate per chamber per collection date (July 29, July 31, and August 3, 2014). The average monthly temperature for the duration of the trials was 20.6°C and the total monthly precipitation was 146.3 mL [36].

On the date of collection, each litterbag was individually placed in a sealed plastic bag and swiftly returned to the laboratory. Subsequently, the contents of each bag were directly placed onto a Petri dish and evaluated using a dissecting microscope. All invertebrates on the leaves and within the litterbag were counted and identified to taxonomic Order (S2 Fig). Richness (order) and abundance of the invertebrate community were calculated for all 90 bags. Eggs, juveniles, and unidentified taxa were incorporated in analyses of abundance but not richness. We measured dry weight of the leaves using the same protocol as above, and leaf litter lost was used a proxy for decomposition following Eq 1:

$$\left(1-\frac{M}{M_{0}}\right)$$ (1)

where *M*_*0*_ is the initial dry weight of the leaves and *M* is the final dry weight of the leaves.

In this study we only capture the early stages of leaf litter loss, though acknowledge that early decomposition dynamics are different from those in the late decomposition period [35]. Nonetheless, in studies that evaluate decomposition for longer periods, the importance of invertebrate exclusion becomes evident after 30 days (e.g. [37]), well within the timeframe of our experiment, enabling us to look for patterns important for the early stages of decomposition.

### Statistical analyses

We used a linear mixed effects model (LMM) that included percent litter lost as the response, predicted by invertebrate exclusion (litterbag treatment), average soil moisture, average air temperature, average soil temperature, and collection date (*n* = 90). Predictive models were fitted with maximum likelihood estimation and included chamber as the random effect using the lme4 package [38]. We evaluated the Variance Inflation Factor (VIF) for each variable, finding that average air temperature and average soil temperature were highly correlated. Once average soil temperature was removed from the model, all VIF values were less than two indicating low collinearity [39]. Starting from a full model which included all possible interactions between average soil moisture, average air temperature, and invertebrate exclusion treatment as predictors, we compared models using the *dredge* function of the MuMIn package [40], selecting the model with the lowest Akaike Information Criterion (AICc) value. We then obtained significance values using a likelihood ratio test that compared the final model to models that iteratively excluded each variable. Post-hoc tests to evaluate differences among invertebrate exclusion treatments were run using the *emmeans* function from emmeans package in R [41]. We employed the same approach to determine the effect of invertebrate exclusion, average soil moisture, and average air temperature on invertebrate abundance and richness, with the sole difference that abundance was log transformed to improve model fit.

We also evaluated changes in community composition, using a Permutational Multivariate Analysis of Variance (PERMANOVA) via the *adonis* function of the vegan package [42], based on Bray Curtis dissimilarities and 9999 permutations. The response was a matrix of the community in each bag predicted by invertebrate exclusion, average air temperature, average soil moisture, as well as all interactions among variables. For community analyses, we pooled samples by size-class treatment for each chamber as there were few individuals collected in any given bag and we expected that the sum across bags within an exclusion treatment within a chamber would be more representative of the community (*n* = 30). The results are visualized using non-metric multidimensional scaling (NMDS) and the *metaMDS* (taxonomic dissimilarity) function of the vegan package [42].

Given that both the invertebrate exclusion treatment and average air temperature affected invertebrate abundance and richness (see Results), we employed a structural equation model to evaluate the direct and indirect effects of average air temperature on decomposition mediated by invertebrate abundance and richness. Using the piecewiseSEM package [43], we constructed a structural equation model that included average air temperature as a predictor of invertebrate abundance, richness, and leaf litter loss in a LMM that included chamber as the random effect. Invertebrate abundance and richness were similarly included as predictors of leaf litter loss. Invertebrate abundance was log-transformed to meet assumptions of normality. Invertebrate abundance and richness were modeled to co-vary.

## Results

**Fig 2:**
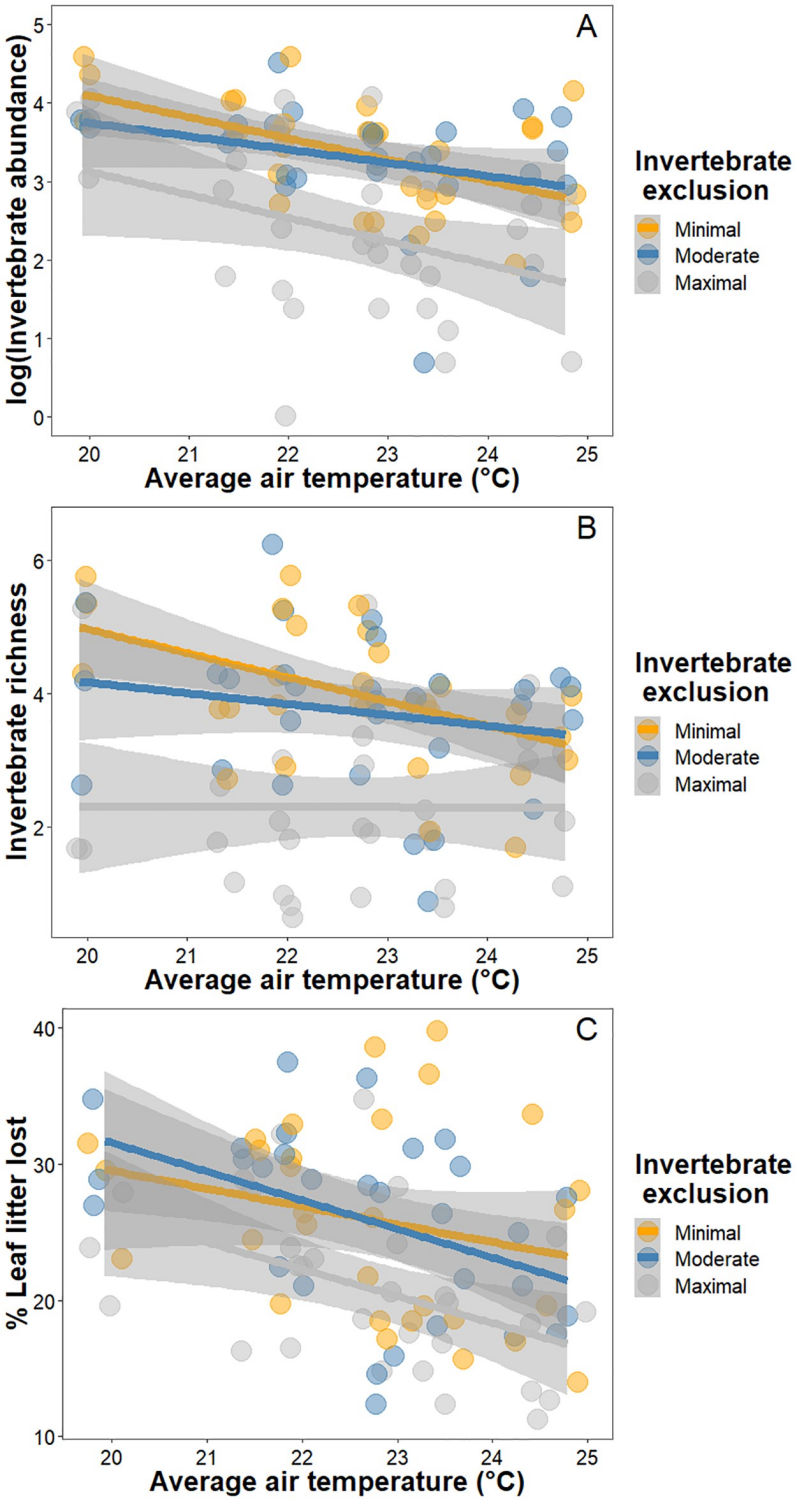
Air temperature and invertebrate diversity and decomposition rates. Average air temperature reduced A) invertebrate abundance, B) invertebrate richness, and C) and percent leaf litter lost in the litterbags.

We collected 3662 invertebrates belonging to 12 taxonomic groups, with the three most abundant groups being the springtails (Collembola), mites (Acari), and booklice (Psocoptera) across the 90 litterbags deployed (S2 Fig). The abundance and richness of the invertebrate community in each bag varied by invertebrate exclusion treatment (χ^2^_2_ = 31.14, *P* < 0.001 and χ^2^_2_ = 40.31, *P* < 0.001, respectively; S2 Fig), with lower abundance and richness in the maximal invertebrate exclusion treatment compared to the moderate and minimal, which did not differ among themselves (S1 Table). There was lower invertebrate abundance and richness with increasing air temperature (χ^2^_1_ = 6.90, *P* = 0.009 and χ^2^_1_ = 5.92, *P* = 0.015, respectively; Fig 2A and 2B). Average soil moisture was not a strong predictor of either invertebrate abundance or richness, not being present in the best-fit models for either response (S2 and S3 Tables).

**Fig 3:**
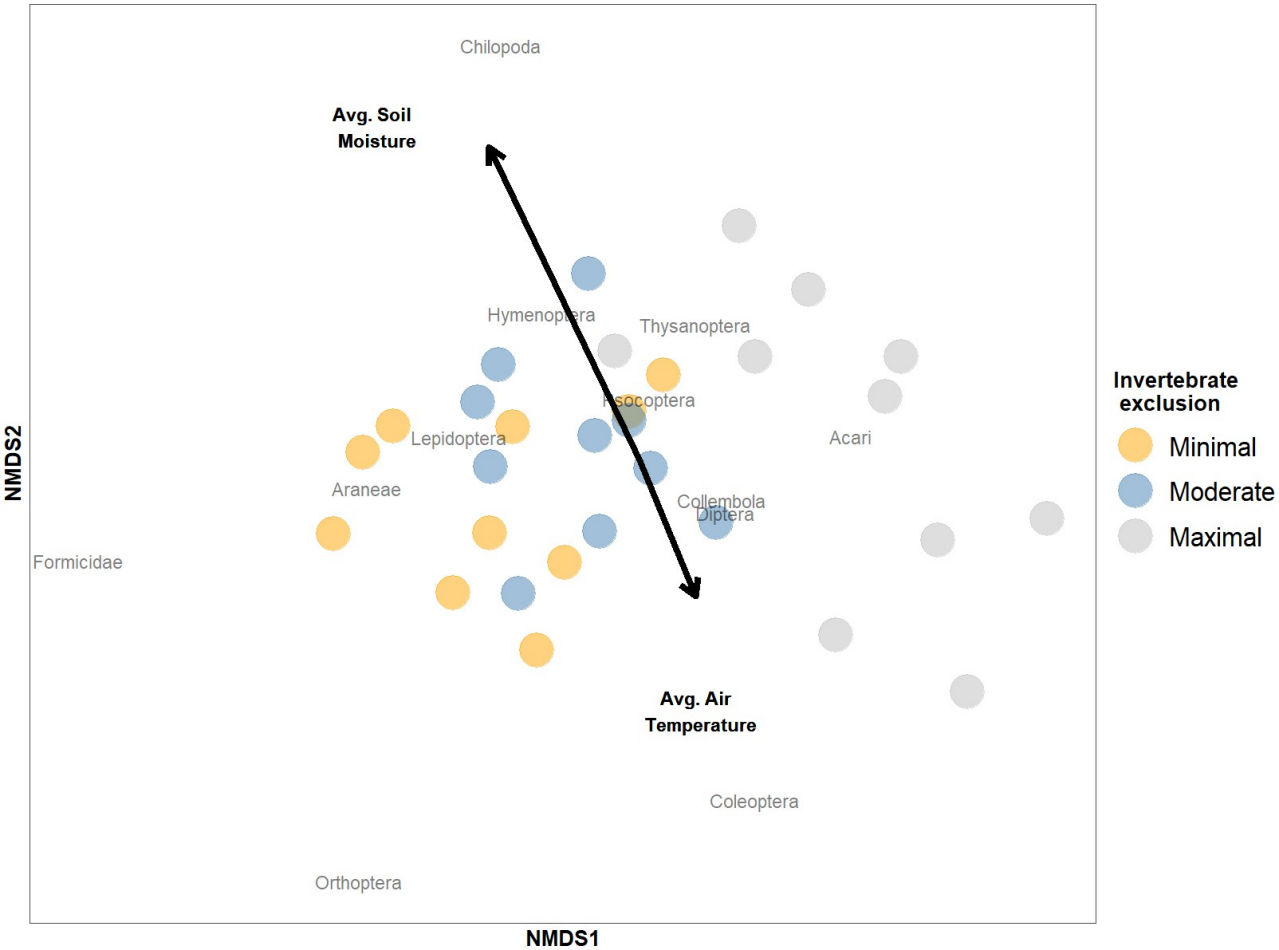
Nonmetric multidimensional scaling (NMDS) plot visualizing the invertebrate community in the litterbags, with invertebrate exclusion treatment separated by color. The communities are aggregated by treatment within chamber (*n* = 30). The direction and length of the arrows indicate the relationship between the environmental factors (average air temperature and average soil moisture) and the community matrix. The NMDS stress is 0.20.

As expected, community composition was affected by the invertebrate exclusion treatment (F_2,29_ = 4.80, R^2^ = 0.23, *P* < 0.001). The invertebrate community similarly was shaped by local environmental factors, namely average air temperature and average soil moisture, as well as their interactions (F_1,29_ = 2.59, R^2^ = 0.06, *P* = 0.048, F_1,29_ = 3.50, R^2^ = 0.08, *P* = 0.016, and F_1,29_ = 4.43, R^2^ = 0.11, *P* = 0.006, respectively; Fig 3). Average air temperature and average soil moisture were not correlated (F_1,8_ = 0.70 *P* = 0.428); increasing air temperature and decreasing soil moisture affected community composition similarly (Fig 3). There were no significant differences in community composition based on interactions between invertebrate exclusion and average air temperature, or invertebrate exclusion and average soil moisture, or the three way interaction among predictors (F_2,29_ = 0.78, R^2^ = 0.04, *P* = 0.610, F_2,29_ = 0.59, R^2^ = 0.03, *P* = 0.783, and F_2,29_ = 0.16, R^2^ = 0.01, *P* = 0.993, respectively).

**Fig 4:**
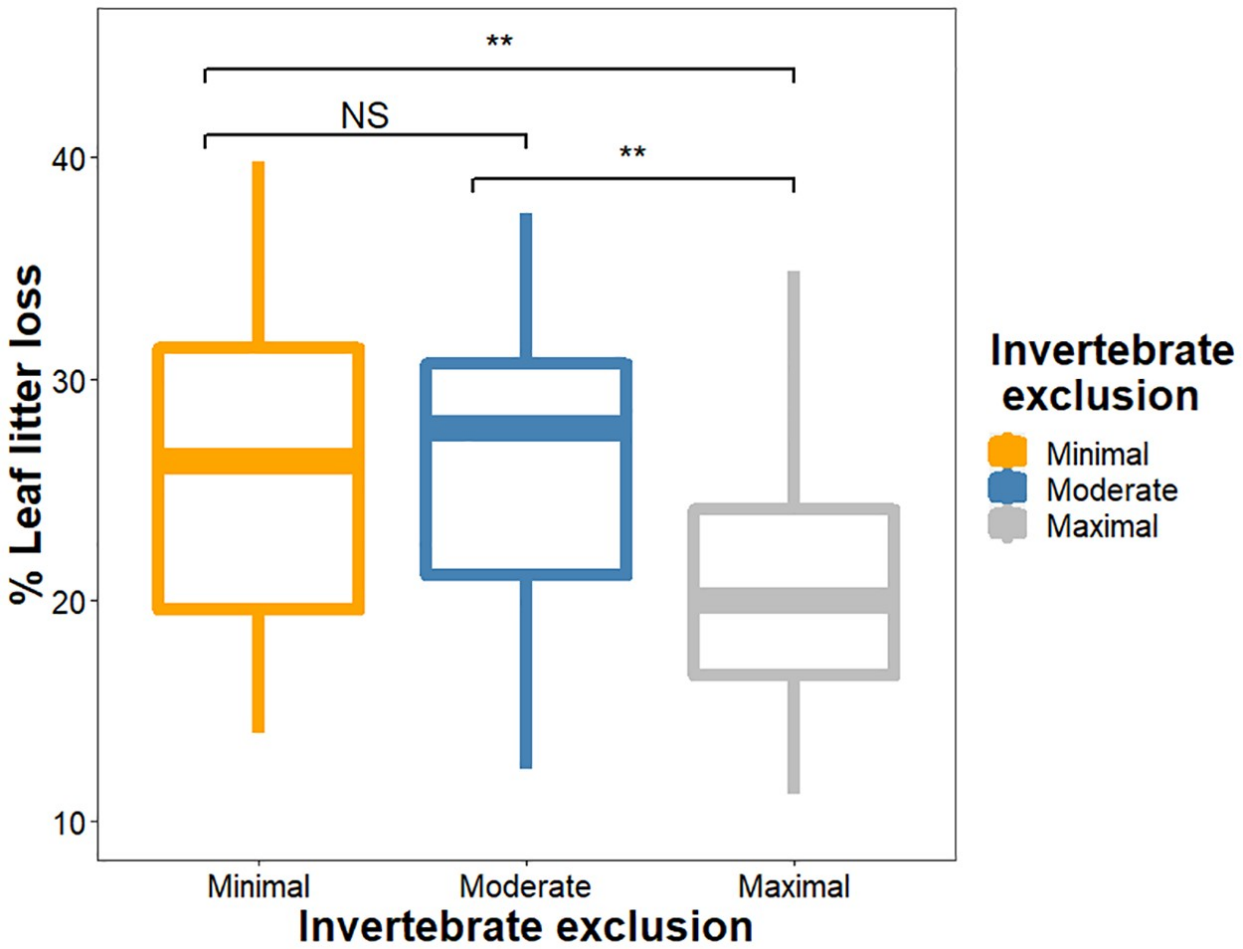
Boxplot showing the relationship between invertebrate exclusion and percent leaf litter lost. Maximal invertebrate exclusion had lower leaf litter loss than minimal and moderate invertebrate exclusion, which in turn did not differ. NS *P* > 0.05 ** *P <* 0.01.

The best model for explaining decomposition rates, based on AICc scores, included an additive relationship between invertebrate exclusion and average air temperature (S4 Table). The invertebrate exclusion treatments affected leaf litter loss (χ^2^_2_ = 12.80, *P* = 0.002), with greater leaf litter loss occurring when invertebrates had ample access to the litter, with maximum invertebrate exclusion having lower leaf litter loss than moderate and minimal invertebrate exclusion, while moderate and minimal invertebrate exclusion did not differ (Fig 4; S1 Table). Simultaneously, we found that average air temperature affected leaf litter loss (χ^2^_1_ = 9.63, *P* = 0.002), with less leaf litter loss occurring in warmer chambers (Fig 2C). Greater temperatures consistently reduced leaf litter loss across treatments, which in turn was lowest in the maximal invertebrate exclusion treatment.

**Fig 5:**
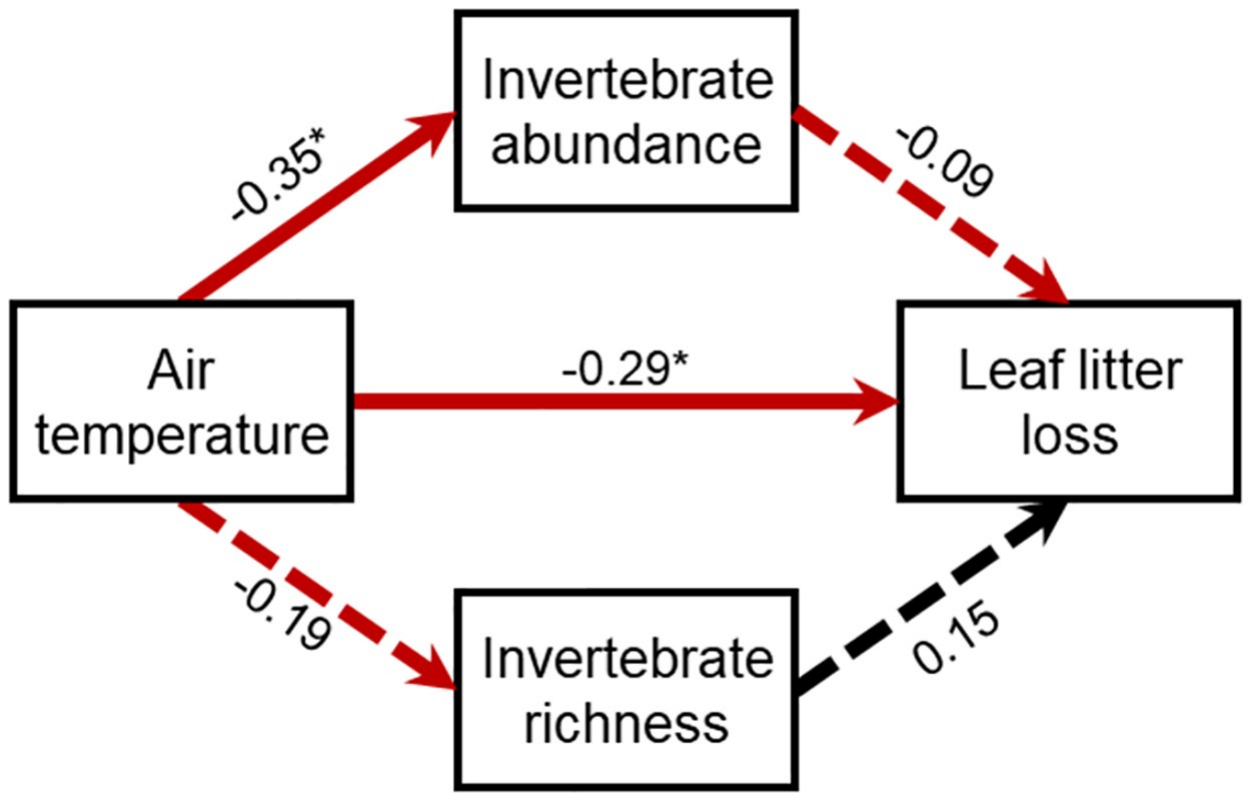
Structural equation model evaluating direct and indirect relationships between average air temperature in the warming chambers and invertebrate abundance, invertebrate richness, and leaf litter loss. The values reported are the standardized path coefficients. Solid lines indicate significant paths (*P* < 0.05), while dashed lines indicate non-significant paths (*P* > 0.05). Red lines indicate negative relationships while black lines indicate positive relationships. The R^2^ for abundance = 0.11 and decomposition = 0.13. * *P* < 0.05.

We constructed a path analysis to evaluate whether reductions in invertebrate abundance and richness explained the effect of average air temperature on leaf litter loss. Surprisingly, there was only a direct link between average air temperature and leaf litter loss, despite average air temperature reducing invertebrate abundance, because in the combined structural equation model invertebrate abundance no longer explained leaf litter loss (Fig 5; S5 Table). Invertebrate richness was neither predicted by average air temperature nor explained leaf litter loss in the path model (S5 Table).

## Discussion

We found that increasing average air temperature reduced invertebrate abundance and richness, and altered soil invertebrate community composition in the Harvard Forest warming chambers (Figs 2 and 3). Average soil moisture did not directly affect invertebrate abundance or richness but did alter the invertebrate community composition. These changes in the invertebrate community affected leaf litter loss, with more leaf litter loss occurring in litterbags housing more invertebrates and more diverse communities (Fig 4). Similarly, average air temperature also affected the early stages of decomposition, with reduced leaf litter loss occurring when the air was warmer (Fig 2C). While average air temperature reduced invertebrate abundance, this reduction was not the mechanism that explained how average air temperature impacted leaf litter loss (Fig 5).

We found that experimentally manipulating invertebrate communities using size exclusion treatments shaped the number of individuals and diversity present in the litterbags (Fig 2A and 2B), ultimately resulting in unique community compositions (Fig 3). Our finding that decomposition was higher in litterbags with greater invertebrate access is strongly supported in the literature [29, 37, 44–46]. Nonetheless, relationships between invertebrate abundance, richness and decomposition are inconsistently linked, as some studies find that decomposition is affected by both factors [25], richness but not abundance [29], neither [37], or attribute differences to biomass rather than richness or abundance [47]. In this study, we found that decomposition varied with invertebrate exclusion based on size class, not simply presence/absence of invertebrates. We speculate that this occurred because the least restrictive litterbags allowed colonization by more individuals, occupying different trophic positions and likely representing a larger suite of functional traits [48]. Under minimal invertebrate exclusion, the communities had greater representation of caterpillars, spiders, and ants (S2 Fig), all of which can contribute to decomposition dynamics either directly (*e*.*g*. caterpillars directly consuming the leaves [49]), indirectly (*e*.*g*. predation by spiders altering behavior of prey and subsequently of the decomposer microbial communities [26, 27]), or both (*e*.*g*. ants directly shredding leaf litter as well as aerating the soil via nest building which indirectly facilitates decomposition [18]). It is important to note that our treatments were not designed to evaluate abundance and richness independently. The inclusion of macroinvertebrates may be particularly important, as large invertebrates can directly and indirectly shape the abiotic environment for the rest of the community [50], which is supported by our finding that litterbags with more diverse and abundant invertebrate communities had greater leaf litter loss.

In our study, warming reduced invertebrate abundance and richness (Fig 2A and 2B), consistent with other studies [51, 52]. Only centipedes and beetles appeared to be markedly influenced by warming (Fig 3), but these groups had very low abundance (S2 Fig), and thus we are unable to ascertain the long-term impacts on diversity. However, not all invertebrate taxa and populations are equally susceptible to warming. For example, Stuble et al. evaluated ant forager abundance and richness in warming chambers, finding that experimental warming increased forager abundance differentially depending on latitude [53]. Similarly, in a common garden experiment, the source of an ant species affected its response to warming, with warming resulting in higher mortality for colonies of *Temnothorax curvispinosus* from cooler locations, a pattern not found for colonies of the same species from warmer locations [54]. Furthermore, we found an interaction between temperature and moisture on community composition, indicating that the effect of warming on community composition varies depending on whether there is low or high moisture content in the environment. Similarly, earthworms burrow further in the ground in warmer conditions, though markedly more in drier conditions, structurally altering the architecture of the system with possible knockdown effects on the soil community [55, 56]. Evaluating the relationship between warming, invertebrate communities, and decomposition at larger scales across diverse natural environments is important for better understanding the impact of climate change.

While our finding lower decomposition rates in warmer chambers contrasts with many other studies which find that decomposition generally increases with temperature [13–16], it is well aligned with a study conducted with the same leaf litter species in the same geographic region [18]. Specifically, Del Toro et al. 2015 evaluated red maple leaf litter loss in mesocosms that were either warmed 3.5°C or 5°C, and either had or did not have ants foraging and nesting. They found that ants decomposed up to 84% less in warmer chambers. Furthermore, they also found a direct negative relationship between warming and leaf litter loss [18]. The authors retained low and stable soil moisture in their experiment across treatments. Similarly, average air temperature and average soil moisture were not correlated in our study and our best-fit model for leaf litter loss did not include soil moisture (S2 Table), suggesting that soil moisture was not a major driver of leaf litter loss in our communities. It is important to note that moisture fluctuations are much stronger in the upper ground layer [31], the most relevant microclimate for the decomposing invertebrate and microbial communities, than deeper in the soil. As such, warming may have affected litter moisture before soil moisture, and thus we do not exclude lower leaf litter moisture in warmer chambers as a possible explanation of the patterns found in our study. Future work elucidating the mechanisms that dictate when warming will increase vs decrease decomposition in the context of a range of invertebrate diversity is warranted.

There were lower decomposition rates under warmer temperatures. This pattern was not driven by invertebrate abundance or richness (to the level of Order), as leaf litter loss was lower in warmer chambers across all three invertebrate exclusion treatments (Fig 2C). Invertebrate access increasing decomposition independently of warming has also been shown in other systems [12, 18]. We found that while warming reduced invertebrate abundance, this reduction was not the mechanism by which leaf litter loss was reduced (Fig 5). Nonetheless, lethal thermal limits were most likely not reached for this community [56] and thus not the likely explanation for the pattern. Instead, we hypothesize that if invertebrates altered the direct effect of warming on leaf litter loss, it would have been a result of changes to invertebrate behavior and/or physiology [57]. Taxa can respond differentially to experimental changes in temperature, with certain species foraging more under warming while others forage less [53], and if important decomposers are less active in warmer environments, nutrient cycling can become impaired. Similarly, experimental warming can induce ants to displace more soil and build deeper nests, and ultimately be less effective decomposers [18]. Even if colonization occurred primarily from within the chamber, which is likely given the relatively limited dispersal ability of soil fauna and the relatively large 5 m chambers used [58], warming may have nonetheless decreased how efficient the remaining invertebrates were at breaking down leaf litter. Pinpointing the most functionally important taxa that are highly susceptible to climate change is an important and time-sensitive future direction.

## Conclusion

Decomposition and subsequent nutrient cycling depends on numerous, often interacting, biotic and abiotic factors. Warming reduced invertebrate abundance, richness, and altered invertebrate community composition. We found that in the early stages of decomposition, greater invertebrate abundance and diversity in the soil community resulted in greater leaf litter loss. This effect was observed regardless of temperature, despite warming consistently reducing decomposition rates across invertebrate exclusion treatments. The direct effect of warming on invertebrate behavior and functional response can mediate how communities will respond to increasing global temperatures. While invertebrates are undoubtedly key players in the decomposition process, our study highlights that climate change can impair the provisioning of this important ecosystem service for both simple and more complex soil invertebrate communities.

## Supporting information

S1 Fig Image of the modified plastic containers used to provide structure across all litterbags. (DOCX)

S2 Fig Invertebrate abundance by taxa across invertebrate exclusion treatments. Invertebrate abundance valued summed across all litterbags of the respective treatment.
(DOCX)

S1 Table Comparison of estimated marginal means (EMM) across invertebrate exclusion treatments for invertebrate abundance, richness, and leaf litter loss. (DOCX)

S2 Table Model comparison table for invertebrate abundance. Output from dredge analysis evaluating invertebrate abundance in the litterbags with environmental and biodiversity metrics as predictors. Models sorted based on AIC score. Variables included within a model are in bold, while *NA* indicates variables that are not included in a given model.
(DOCX)

S3 Table Model comparison table for invertebrate richness. Output from dredge analysis evaluating invertebrate richness in the litterbags with environmental and biodiversity metrics as predictors. Models sorted based on AIC score. Variables included within a model are in bold, while *NA* indicates variables that are not included in a given model.
(DOCX)

S4 Table Model comparison table for leaf litter loss. Output from dredge analysis evaluating decomposition (leaf litter loss as response) with environmental and biodiversity metrics as predictors. Models sorted based on AIC score. Variables included within a model are in bold, while *NA* indicates variables that are not included in a given model.
(DOCX)

S5 Table Results from the structural equations model (Fig 5). (DOCX)
