# Supplementary material for: Increasing temperatures reduce invertebrate abundance and slow decomposition
Source: PLoS One. 2021 Nov 10;16(11):e0259045. doi: 10.1371/journal.pone.0259045 (PMC8580216; doi:10.1371/journal.pone.0259045)

**S2 Fig. Invertebrate abundance by taxa across invertebrate exclusion treatments.** Invertebrate abundance valued summed across all litterbags of the respective treatment.


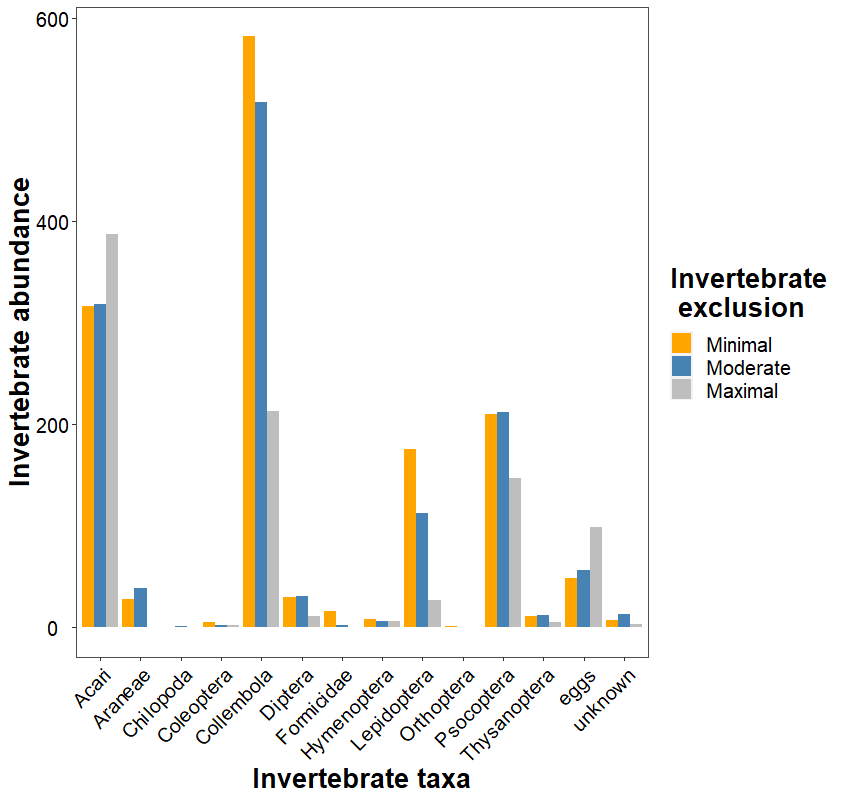

Supplement: S2 Fig — Invertebrate abundance valued summed across all litterbags of the respective treatment. (DOCX) [file pone.0259045.s002.docx]
