# Supplementary material for: Increasing temperatures reduce invertebrate abundance and slow decomposition
Source: PLoS One. 2021 Nov 10;16(11):e0259045. doi: 10.1371/journal.pone.0259045 (PMC8580216; doi:10.1371/journal.pone.0259045)
Supplement: S1 Table — (DOCX) [file pone.0259045.s003.docx]

**S1 Table. Comparison of Estimated Marginal Means (EMM) across invertebrate exclusion treatments for invertebrate abundance, richness, and leaf litter loss.**
