# Supplementary material for: Increasing temperatures reduce invertebrate abundance and slow decomposition
Source: PLoS One. 2021 Nov 10;16(11):e0259045. doi: 10.1371/journal.pone.0259045 (PMC8580216; doi:10.1371/journal.pone.0259045)
Supplement: S4 Table — Output from dredge analysis evaluating decomposition (leaf litter loss as response) with environmental and biodiversity metrics as predictors. Models sorted based on AIC score. Variables included within a model are in bold, while NA indicates variables that are not included in a given model. (DOCX) [file pone.0259045.s006.docx]

**S4 Table. Model comparison table for leaf litter loss.** Output from dredge analysis evaluating decomposition (leaf litter loss as response) with environmental and biodiversity metrics as predictors. Models sorted based on AIC score. Variables included within a model are in bold, while *NA* indicates variables that are not included in a given model.
