# Supplementary material for: Increasing temperatures reduce invertebrate abundance and slow decomposition
Source: PLoS One. 2021 Nov 10;16(11):e0259045. doi: 10.1371/journal.pone.0259045 (PMC8580216; doi:10.1371/journal.pone.0259045)
Supplement: S5 Table — (DOCX) [file pone.0259045.s007.docx]

**S5 Table. Results from the structural equations model (see also Fig 5).**
